# Growth, development, and life history of a mass-reared edible insect, *Gryllodes sigillatus* (Orthoptera: Gryllidae)

**DOI:** 10.1093/jee/toaf073

**Published:** 2025-04-18

**Authors:** Jacinta D Kong, Marshall W Ritchie, Émile Vadboncoeur, Heath A MacMillan, Susan M Bertram

**Affiliations:** Department of Biology, Carleton University, Ottawa, Ontario, Canada; Department of Biology, Carleton University, Ottawa, Ontario, Canada; Department of Biology, Carleton University, Ottawa, Ontario, Canada; Department of Biology, Carleton University, Ottawa, Ontario, Canada; Department of Biology, Carleton University, Ottawa, Ontario, Canada

**Keywords:** insect rearing, insect agriculture, performance, alternative protein production, allometry

## Abstract

Edible insects offer a viable alternative protein source to help meet the protein demands of a growing population. Optimizing insect mass-rearing for food and feed production depends on an understanding of insect life history. However, supporting data on growth, development, and reproduction from hatch to adulthood is often not available for many farmed insects, such as the decorated cricket (*Gryllodes sigillatus* Walk.). Here, we describe the life history of *G. sigillatus* from hatch to adulthood at 30 °C for traits relevant for mass-rearing and colony management. Female crickets first reached adulthood after 29 d and weighed 292.0 mg ± 74.09 mg, and male crickets first reached adulthood after 35 d and weighed 200.96 mg ± 34.51 mg. Crickets had 7 nymphal instars most characterizable by head width. Sex was identified by the development of ovipositors in females, and wings in both sexes. Crickets oviposited 56.74 ± 31.77 eggs every 48 h over 30 d and eggs hatched after 10.6 ± 0.5 d. This information provides the foundation to start and manage a cricket colony, to conduct research on life history and performance, and to facilitate practitioners to make informed decisions about rearing practices or identify arising issues. We highlight ways that a fundamental understanding of cricket biology can be informative for optimizing cricket growth, reducing variability in yield, and informing future precision farming practices.

## Introduction

Insects have been economically and culturally important as food to many communities around the world (Raheem et al. 2019). In recent years, intensive commercial insect farming has gained attention as one means to meet the protein demands of an increasing global population (van Huis 2020). Insect farming is considered an alternative to conventional agriculture that aligns with the UN’s Sustainable Development Goals (UN 2015, Liceaga et al. 2022). Raising insects uses less space and water than conventional agriculture, is estimated to produce fewer greenhouse gases, and can be integrated within sustainable practices, such as a circular economy (Chavez 2021, van Huis et al. 2021). Research and development play a crucial role in ensuring the edible insect industry develops effectively and sustainably by generating data that can inform, improve and optimize farming practices (Van Peer et al. 2024). Acquiring and implementing these data requires collaborative partnerships between academics, industry, and government agencies (Tomberlin et al. 2022, Robinson et al. 2024). Through these partnerships, research outcomes can be translated into commercial practice and can be used to quantitatively evaluate, refine, and adapt practices (Morales-Ramos et al. 2024).

Data-driven insect mass-rearing depends on fundamental biological information that draws expertise from agriculture and animal science, entomology, ecology, and evolution. Insects used in the edible insect industry are typically small, rapidly reproducing with short generation times and dense populations, and are amenable to manipulations of factors like temperature, diet, and humidity (Davidowitz 2021, Hawkey et al. 2021). Thus, the yield and productivity of commercial insect farms are highly dependent on species-specific biological traits that can be targets for research and development. Individual-level traits, such as instar progression, body size and mass throughout ontogeny, reproductive output and egg development, drive population dynamics that will determine the success of a colony and the resulting product yield. Variable yield among harvests is a problem that can emerge even when rearing conditions are unchanged (Moore and Martin 2019, Mutamiswa et al. 2023). Variability in yield generates uncertainty in the supply of insect products, which can slow the rate of industrial development needed for a profitable and sustainable industry (Larouche et al. 2023). One solution to this problem is to use a species-centric data-driven approach that allows for fine-scale optimization of rearing practice and for troubleshooting production issues. Examples of optimization studies in mass-reared insects include the effects of temperature for rearing (Chia et al. 2018, Mamai et al. 2018, Chen et al. 2022), diet preferences (Morales-Ramos et al. 2020), and reproduction and husbandry (Hoc et al. 2019). Failure to incorporate biological knowledge or make information publicly available can hinder efforts to troubleshoot production industry-wide (Larouche et al. 2023). However, the advantages of a data-driven farming approach also underpin the challenges of this approach; the requirements or costs for obtaining the data necessary to troubleshoot where and when in production unwanted variability in yield is generated can be prohibitively high (Davidowitz 2021). This can be a problem for insect producers for whom research and development is not a priority and thus do not possess the facilities, time, money or expertise to troubleshoot production. To this end, partnerships between academia and industry are important because academics typically have the facilities and expertise to prioritize the necessary research and development and can assist in troubleshooting (Tomberlin et al. 2022).

Several true crickets (Orthoptera: Gryllidae) are farmed for consumption globally (Magara et al. 2021). In North America, the decorated cricket *Gryllodes sigillatus* (Walker) and the house cricket *Acheta domesticus* L. are the 2 main commercially farmed species (Larouche et al. 2023). *Acheta domesticus* has been widely used as a model species in entomology and more recently in an edible insect farming context because of its widespread use, leading toward calls for standardized data collection (Morales-Ramos et al. 2024, Van Peer et al. 2024). In contrast, *G. sigillatus* has received less attention particularly in a farming context despite its popularity as a farmed species and its long history of use as a model cricket species. There is a wealth of knowledge about the life-history traits of adult *G. sigillatus* including digestive morphology (Biagio et al. 2009, Ritchie et al. 2024), immunity (Gershman et al. 2010), diet (Rapkin et al. 2018), adult lifespan (Archer et al. 2012), and reproductive effort to name a few (Bateman et al. 2001, Ketola and Kotiaho 2010, Okada et al. 2011). However, as mass-rearing is not the focus of these studies, we are missing a comprehensive description of the growth and development of *G. sigillatus* from hatch to adulthood that is directly relevant to farming (Muzzatti et al. 2022). Here, we aimed to fill this knowledge gap by describing the instar progression, growth, development, and egg production of a farmed strain of *G. sigillatus*. This information will allow practitioners to track development, design experiments aiming to optimize rearing protocols for this species, and potentially identify sources of production variation.

## Methods

### Colony Care and Husbandry

We maintained a colony of *G. sigillatus* at Carleton University, Ontario, Canada to ensure a consistent supply of individuals for several experiments. Separate cohorts of eggs from this colony were used to quantify life-history characteristics in this study. These individuals were originally sourced as eggs from Entomo Farms, Ontario, Canada that have been mass-rearing a population for food and feed for over a decade. The colony was maintained at 30 °C (30.24 ± 1.43 °C, mean ± standard deviation), 30% relative humidity (R.H.) (30.88 ± 11.01%), and a 14:10 h Light:Dark photoperiod. Room temperature and R.H. were monitored using data loggers (IBS-TH2, Inkbird, Guangdong, China). All crickets were provided food (cricket feed mix of soy, corn, fishmeal, and micronutrients, 1:0.94 Protein: Carbohydrate, Campbell Feed Mill, Ontario, Canada), water as wet paper towels in a vial, and egg carton as shelter. The colony consisted of ten 5.2 L plastic containers (22.9 × 15.2 × 15.2 cm, L × W × H) containing approximately 200 crickets in each container until instars 6 to 7, then the number of crickets was reduced to 20 per container to minimize potential effects of high rearing density. As part of the continued colony maintenance, adult crickets were allowed to oviposit into moist coco peat for 48 h. The peat was collected and incubated under the same conditions as the parents until the eggs within hatched. Egg development time was taken as the time between when the peat was added to the adult colony and the day the first hatched nymph was observed. The hatched nymphs were either used to start a cohort to be used in experiments or another generation of the colony. Each container of the next generation of the colony was seeded from 2 parental containers to allow genetic mixing.

### Nymph Growth, Development, and Morphometrics

**Fig. 1.**
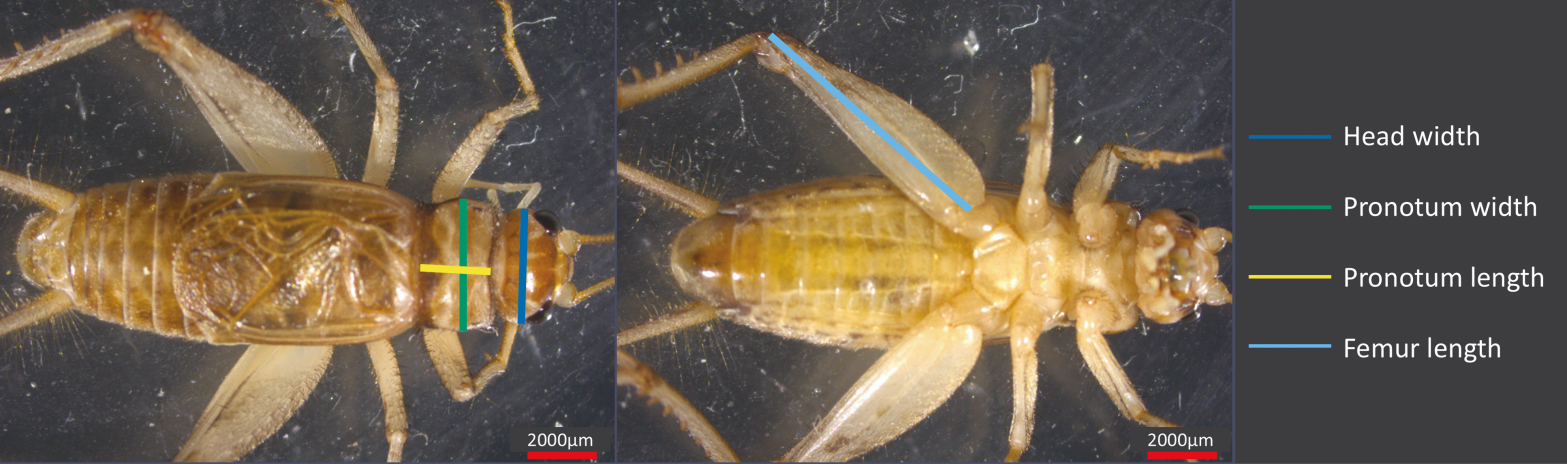
Morphometric landmarks for *Gryllodes sigillatus* from dorsal (left) and ventral (right) photographs. Adult male shown. Images were analyzed in ImageJ. Photos by M. W. Ritchie.

To capture instar progression, a cohort of ~500 crickets from the colony were housed in a plastic bin (68 L, 61 × 41 × 32 cm) under colony conditions. Crickets were randomly sampled every 2 to 3 d for 7 wk (355 individuals in total). Individual crickets were anesthetized with CO_2_ for 15 s, weighed to obtain wet mass, and then photographed under a dissecting microscope (Stemi 508 with an axiocam 105 color, Zeiss, Oberkochen, Germany). Dorsal and ventral photographs were obtained for each individual. Sex if possible was determined through visual inspection of the photo, and the body parts were measured in Fiji (ImageJ) v2.3.051 (Schindelin et al. 2012). Body parts measured were maximum head width (distance between the 2 outside edges of the eyes), maximum pronotum width, maximum pronotum length, and maximum femur length (**Fig. 1**). A subset of these crickets were then frozen at −20 °C (*n* = 265), then desiccated in an oven at 60 °C for at least 72 h (Precision Scientific, IL, USA), and then weighed using a microbalance (ME5, Sartorius, Göttingen, Germany) to determine dry mass and water content.

### Fecundity

A cohort of ~100 crickets originating as eggs from the colony were kept together from hatch until sex could be easily determined (sixth instar), then assigned separate containers for males and females. When crickets had reached adulthood (39 d since first nymphs hatched), male and female pairs (*n* = 25) were set up and kept under colony conditions (30 °C setpoint; 31.15 ± 1.73 °C and 33.56 ± 4.95% R.H., mean ± standard deviation). Temperature and relative humidity were monitored using a data logger on the same shelf as the crickets for the duration of the experiment (IBS-TH2, Inkbird, Guangdong, China). Crickets were weighed (AB135-S, Mettler Toledo, ON, Canada) to obtain adult mass then randomly assigned mating pairs. Mating pairs were housed in a food container (710 ml, 190 × 127 × 70 mm) with a piece of egg carton for shelter and fresh feed in an ink cap (17 mm diameter). Fresh feed was replaced every week. A small container filled with marbles and water (96 ml, 70 mm diameter × 30 mm) provided water and an oviposition substrate. Water was chosen as an oviposition substrate as crickets will oviposit in water if provided no other substrate and it is easier to remove eggs from water for counting than from sand or peat. Using water introduces a risk that crickets may drown, but marbles help to mitigate this risk. Two pairs of the original 25 pairs were excluded from analysis because one pair died 2 d into the experiment without ovipositing and one pair died after ovipositing a single batch of egg on day 6, giving 23 pairs for analysis. In both cases, the female had drowned. Eggs were decanted from the marbles and water every 2 d, at which time the water was refilled. Photographs of egg batches were used to count large batches. Males were removed if the paired female died. Females were retained if the paired male died (5 females at the end of the experiment had a partner that died earlier). Eggs were collected until all surviving crickets had oviposited eggs for 30 d from the first batch of oviposition being observed (38 d in total since the start of pairing). The delay in first oviposition was calculated as the difference between when the first batch of eggs was oviposited and when crickets were allocated to mating pairs. Fecundity was calculated as the mean number of eggs oviposited every 48 h over the 30-d period since first oviposition for each pair. Pairs where the female had died before 30 d (*n* = 5 of 23) were retained in analysis.

### Statistical Analysis

Data were analyzed in R v4.3 (R Core Team 2023). To assess autocorrelation amongst closely related traits, we calculated Pearson correlation coefficients for pairwise morphometric traits, pooling sex (*n* = 355). To assess overall relationships between morphological traits and instar, we fitted linear regressions to Log_10_ transformed morphological traits, pooling sex, and treating instar as a continuous variable. Morphological traits were Log_10_ transformed to meet the assumption of linearity. To assess for sex-specific differences in morphological traits, a separate linear regression was fitted to the subset of data for instars 5 to 7 and the adult stage as these are the data when sex could be identified. Morphological traits were not Log_10_ transformed as these data met the assumptions of linearity. To assess for sexual size dimorphism in wet mass, we treated instar as a categorical variable in an ANOVA and used Tukey’s Honest Significant Differences as a post-hoc test.

To account for heteroskedasticity in wet mass as crickets grow, we fitted a weighted logistic growth curve to wet mass over time with a power variance function for weighting wet mass using *nlme* v3.1 (Pinheiro and Bates 2009). An initial likelihood ratio test showed the weighted logistic regression provided a better fit to the data than the unweighted regression (Likelihood ratio = 982.7, *P* < 0.001), which showed heteroskedasticity in standardized residuals. The relationship between wet mass and instar was Log_10_ transformed to meet the assumption of linearity. Mass and instar were not included as predictor variables in the same model to avoid autocorrelation (*r* = 0.95). We used linear regression to assess the relationship between untransformed wet and dry mass.

Fecundity was analyzed with a mixed-effects model using *nlme* with time standardized to first oviposition as a fixed continuous variable and pair as a random variable (*n* = 23). Time was standardized to account for the variation in when oviposition starts among pairs. The effect of time on fecundity was analyzed using a Wald test (Type III ANOVA) and the random effect of pair on fecundity was tested using a likelihood ratio test (Analysis of Deviance) against the reduced fixed effects model. The effect of parental mass on pair fecundity was analyzed for males and females separately using linear regression.

## Results

### Egg Development

The first nymphs emerged at similar times among independent containers (**Table 1**). Emergence during the first 24 h was minimal and peaked over the next 48 h. Emergence reduced 3 d after the first nymphs emerged and we observed this trend across independent containers even though emergence over time was not formally quantified.

**Table 1.** Life-history traits of mass-reared crickets, *Gryllodes sigillatus*. Crickets were reared at 30 °C.

Trait	Mean value ± standard deviation (sample size)
Egg development time (days)	10.6 ± 0.5 (20)
Mean time to first oviposition (days)	5.66 ± 1.67 (23)
Fecundity (Mean number of eggs per 48 h per female within 30 d of oviposition)	56.74 ± 31.77 (23)
Dry mass at hatch (24 h old nymphs, mg)	0.0313 ± 0.016 (9)

### Morphometrics

**Fig. 2.**
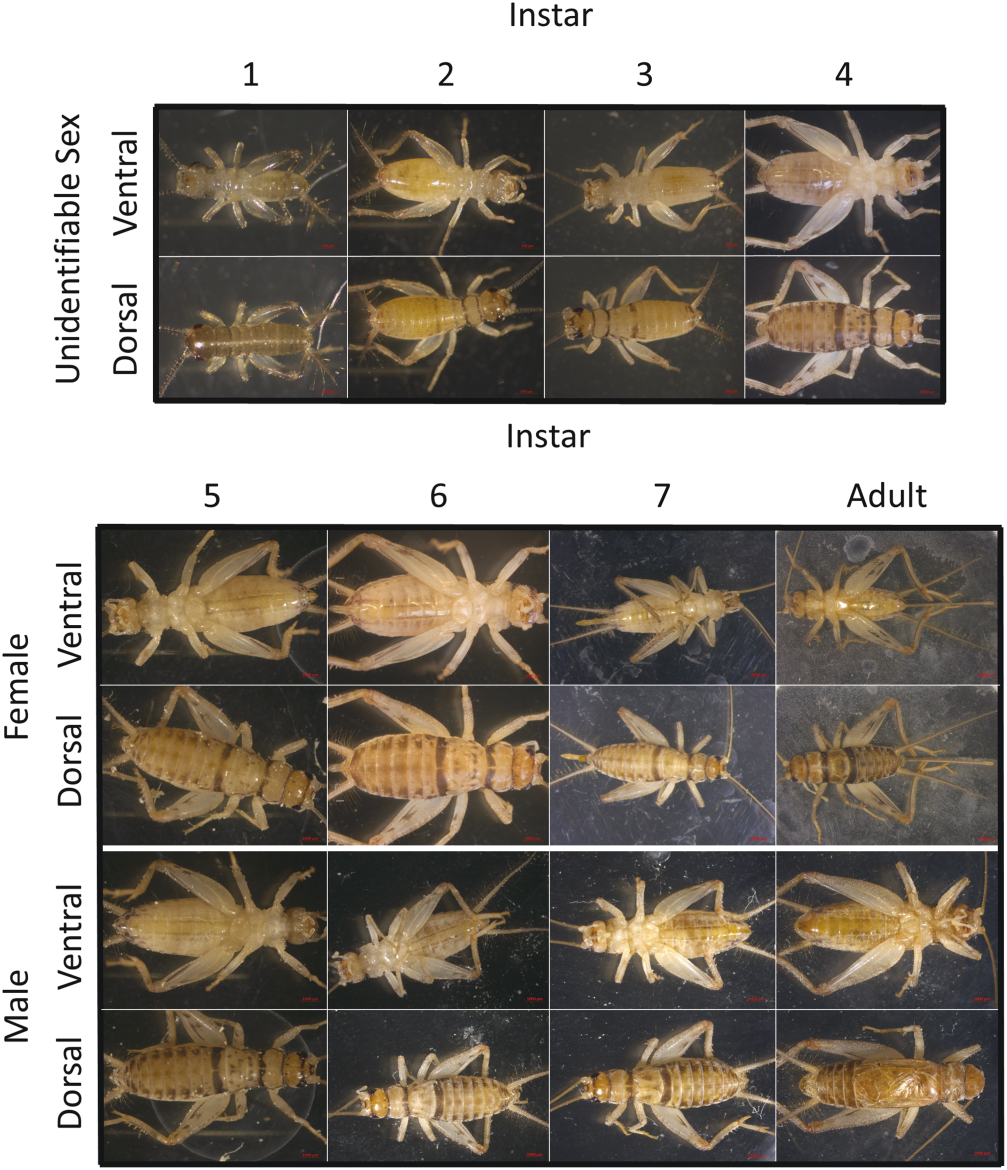
Ventral and dorsal photos of representative *Gryllodes sigillatus* crickets for each instar (columns) and sex (Unidentifiable, Female and Male). Photos by M. W. Ritchie.

The life cycle of *G. sigillatus* consisted of 7 nymphal instars and an adult stage that are identifiable by morphometric characteristics (**Fig. 1**). Crickets molted every 3 to 4 d between instars 1 to 5 in the first 2 wk. These instars are identifiable in photographs by their relative size and shape but lacked distinguishing characteristics easily visible to the naked eye (**Fig. 2**).

**Fig. 3.**
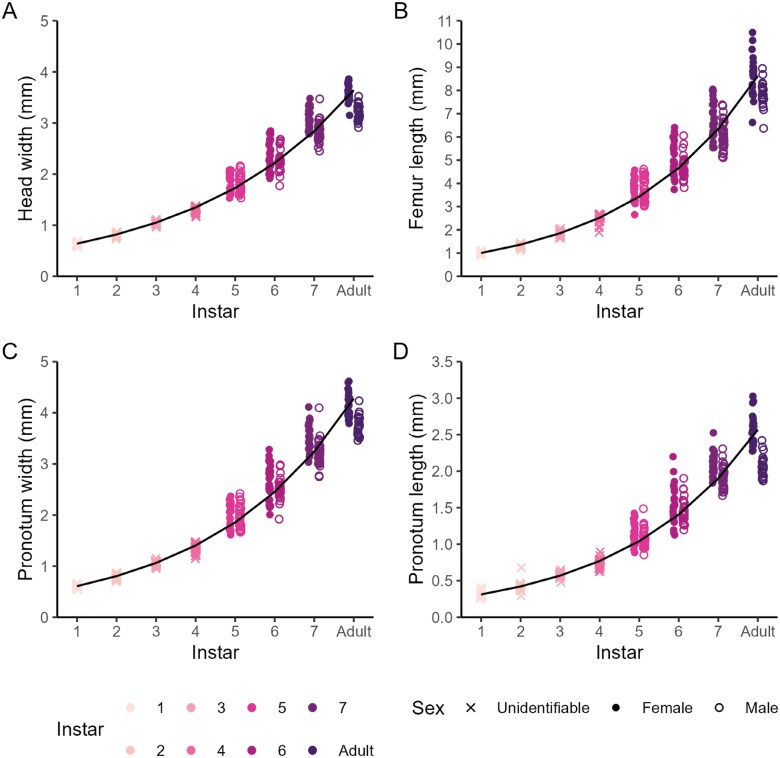
A) Head width, B) Femur length, C) Pronotum width, and D) Pronotum length for each instar of *Gryllodes sigillatus* (colors, n = 355). Crosses denote juvenile crickets of unidentifiable sex (instars 1 – 4), closed circles denote female crickets (instars 5 – 7 & Adult), and open circles denote male crickets (instars 5 – 7 & Adult). Solid black line indicates the fitted linear regression to Log_10_ transformed traits, pooling sex (**Table 2**).

Sex was first identifiable at instar 5 when ovipositors first appeared in females. At instar 5, the ovipositor was not easily visible without careful inspection or magnification as it does not extend beyond the abdomen (**Fig. 2**). Ovipositor length of instar 6 and 7 females varied among individuals of the same instar but were visually distinctive and distinct from a fully formed ovipositor of adults. Wing buds on females first appeared at instar 7, while they appeared on males at instar 6. The size and presence of wing buds were variable, particularly in females but were distinctive for males of instar 6 and 7 and were absent in instar 5 males. Crickets reached reproductive maturity at the adult stage, characterized by fully formed ovipositors in females and wings in males. Males started signaling acoustically for mates at the adult stage. Instars 6 until adult were easily identifiable by these sex-specific morphological characteristics but were less easily distinguished by size and shape alone as data clusters of instars overlapped (**Figs. 2 and 3**).

**Table 2.** Regression equations and coefficient of determination, *R*^2^ (%), for relationships shown in Figs 3 and 4.

Figure panel	Trait	Equation	*R* ^2^ (%)
Fig. 3A	Head width (mm) by instar	Log_10_(Head width) = 0.11 × Instar—0.30	97.59
Fig. 3B	Femur length (mm) by instar	Log_10_(Femur length) = 0.13 × Instar—0.13	97.52
Fig. 3C	Pronotum width (mm) by instar	Log_10_(Pronotum width) = 0.12 × Instar—0.34	97.57
Fig. 3D	Pronotum length (mm) by instar	Log_10_(Pronotum length) = 0.13 × Instar—0.64	96.41
Fig. 4A	Wet mass (mg) over time	$Wetmass=\frac{237.61}{1+e^{\left(\frac{25.38-time}{4.35}\right)}}$	-
Fig. 4B	Wet mass (mg) by instar	Log_10_(Wet mass) = 0.35 × Instar—0.30	95.57
Fig. 4C	Wet mass (mg) by dry mass (mg)	Wet mass = 3.17 × Dry mass + 8.72	97.10

Transformed morphometric traits followed tight linear correlations with instar where data clustered at each instar (**Fig. 3**). Changes in morphometric traits with increasing wet mass were best described by a power function (**Supplementary Fig. S1**, **Supplementary Table S1**) and all morphometric traits were highly correlated with each other (*r* = 0.97 to 1, **Supplementary Fig. S2**). Head width was the most reliable single predictor of instar as it increased stepwise throughout ontogeny and was relatively invariant within an instar (**Table 2**). Head width also positively correlated with mass and had the highest coefficient of determination of all the morphological traits we measured. As females were larger overall than males, females had larger head widths than males and larger increases over time (Instar × Sex: t_227_ = − 4.19, *P* < 0.001).

### Growth Curve and Dry Mass

**Fig. 4.**
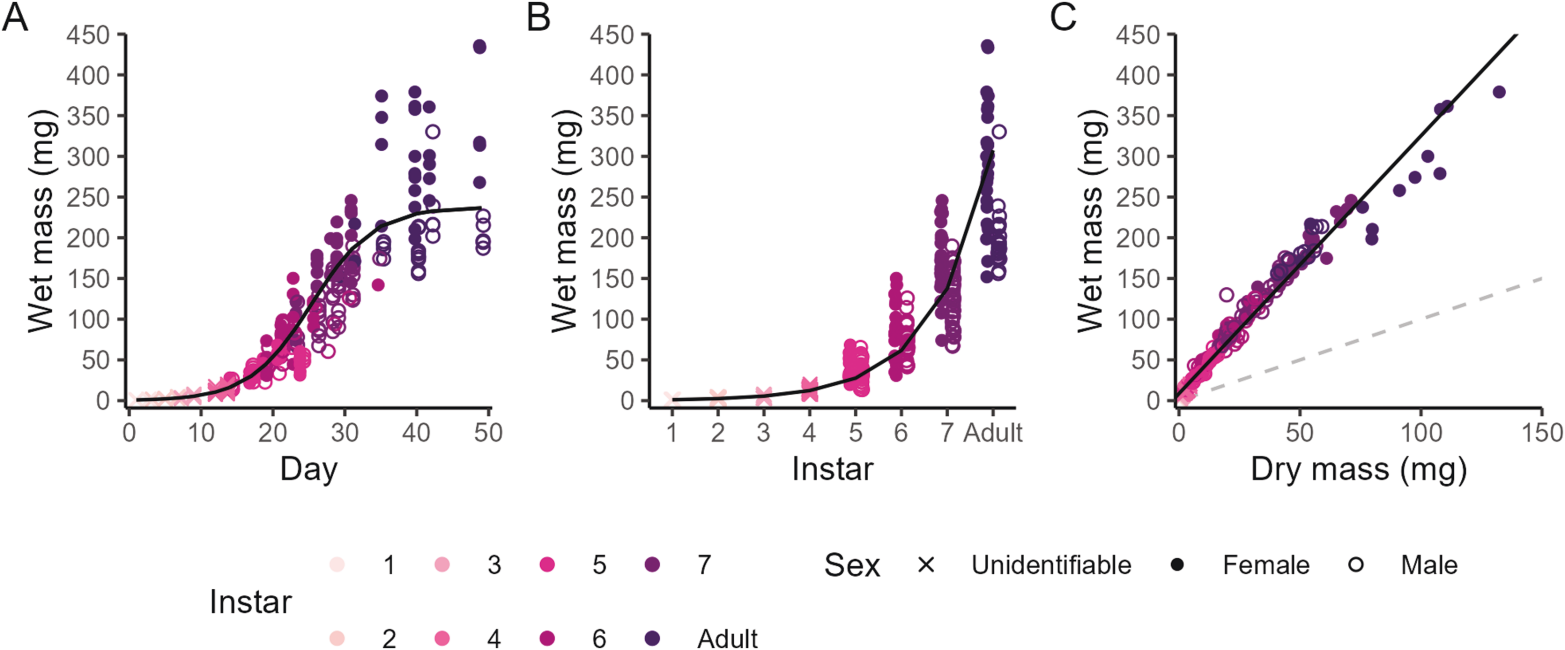
Cricket (*Gryllodes sigillatus*) mass for A) wet mass over time (n = 355), B) wet mass by instar (colors), and C) wet mass against dry mass (mg, n = 265) Crosses denote juvenile crickets of unidentifiable sex, closed circles denote female crickets, and open circles denote male crickets. Dashed gray line indicates the identity line and the solid black line indicates the fitted regressions (**Table 2**).

Increases in wet mass followed a sigmoidal pattern throughout ontogeny (**Table 2**, **Fig. 4A**) and increased exponentially with instar progression (**Table 2**, **Fig. 4B**). Female crickets first reached adulthood after 29 d and weighed 292.0 mg ± 74.09 mg, and male crickets first reached adulthood after 35 d and weighed 200.96 mg ± 34.51 mg. Dry mass had a tight relationship with wet mass (**Table 2**, **Fig. 4C**). Sexes differed in wet mass at instars 7 and at the adult stage, but not earlier (Tukey’s HSD *P* < 0.05 only for instar 7 and adults). Females were heavier and had larger increases in mass over time than males (**Supplementary Fig. S3**, ANOVA, Instar × Sex: *F*_3,223_ = 16.6, *P* < 0.001). Crickets had a water content of 75.7% on average across all instars, and adult females had a lower water content than adult males (**Table 3**).

**Table 3.** Wet and dry mass (mg) and water content (%) of crickets (*Gryllodes sigillatus*) throughout ontogeny.

Instar	Sex	Mean wet mass (mg ± standard deviation)	Mean dry mass (mg ± standard deviation)	Mean water content (%± standard deviation)	Sample size
1	Unidentifiable	1.01 ± 0.34	0.14 ± 0.11	88.16 ± 6.93	19
2	Unidentifiable	2.84 ± 1.18	0.48 ± 0.16	79.59 ± 3.15	11
3	Unidentifiable	5.06 ± 1.88	1.09 ± 0.48	78.97 ± 2.76	24
4	Unidentifiable	12.13 ± 3.01	2.87 ± 0.84	76.11 ± 3.67	31
5	Female	40.2 ± 14.39	10.37 ± 3.93	75.61 ± 2.63	20
5	Male	34.73 ± 13.48	8.51 ± 3.76	75.66 ± 3.47	33
6	Female	80.08 ± 32.74	19.64 ± 9.51	75.28 ± 3.02	24
6	Male	76.66 ± 19.57	20.57 ± 6.85	73.78 ± 4.55	22
7	Female	173.10 ± 39.42	49.29 ± 13.18	71.69 ± 2.38	26
7	Male	125.45 ± 34.29	32.6 ± 10.84	73.75 ± 2.96	31
Adult	Female	292.00 ± 74.09	87.12 ± 26.68	66.78 ± 4.5	13
Adult	Male	200.96 ± 34.51	51.01 ± 6.34	72.45 ± 1.66	10

### Fecundity

**Fig. 5.**
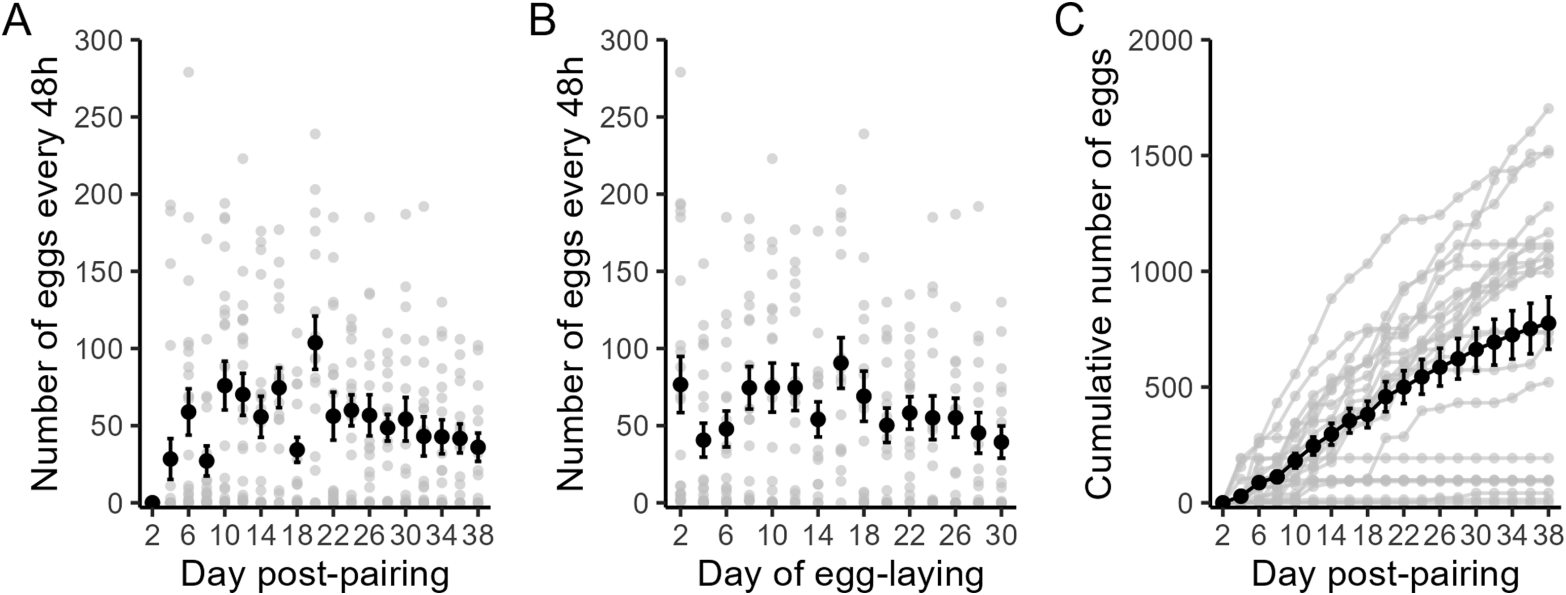
Fecundity (number of eggs every 48 h) of male and female pairs over time from A) when crickets (*Gryllodes sigillatus*) were paired until the end of the experiment, and B) from first oviposition until 30 d had passed. C) Cumulative number of eggs for each pair. Grey points indicate each batch of eggs from each pair (n = 23) and black points and bars represent the mean and standard error across pairs.

We observed some crickets mated immediately after pairing but we did not quantify mating behavior. The first eggs were oviposited between 2 and 10 d after pairing (median 6 d, **Table 1**). Fecundity of a pair (mean number of eggs oviposited in each 48 h period within 30 d of the start of oviposition, *n* = 23) ranged from 0 to 101.67 eggs (**Table 1**, **Fig. 5**). One pair did not oviposit any eggs for the duration of the female’s lifespan (18 d post pairing). The maximum number of eggs oviposited each 48 h period ranged from 0 to 279 eggs across the mated pairs. Not all pairs oviposited eggs every 2 d (**Fig. 5C**; cumulative increases in the number of eggs oviposited was not linear). Overall mean fecundity was 56.74 ± 31.77 eggs in 48 h. Overall mean fecundity did not change over time (*t*_1, 250_ = −1.95, *P* = 0.052) and trends in fecundity varied among pairs (χ^2^ = 13.62, *P* < 0.001). Females continued to oviposit eggs after their male partner had died. Damaged ovipositors were observed in some females over time and most females appeared to stop ovipositing after this damage, but one female was observed to oviposit with a damaged ovipositor. Parental mass was not associated with pair fecundity for males (**Supplementary Fig. S4A**, *t*_1, 21_ = 0.79, *P* = 0.44) or females (**Supplementary Fig. S4B**, *t*_1, 21_ = 1.32, *P* = 0.20).

## Discussion

The life-history data we report fills a knowledge gap about fundamental *G. sigillatus* biology. Although our study concerns one population, comprehensive life-history data from hatch to adulthood, including instar, was not previously available for this species. This absence of information limited our initial ability to design experiments targeting the growth and development of this species for mass-rearing and to manage a colony, which provided the rationale for this study. Here, we sampled a colony of *G. sigillatus* to characterize life history throughout development and into adulthood. This resolution of data (individuals collected every 2 to 3 d) is time and labor intensive and requires expertise and specialist equipment to collect that may be beyond the capacity for an insect farm that does not invest in their own research facilities. Thus, life-history data are not always publicly available despite their value for optimizing farming practices and troubleshooting variation in yields (Davidowitz 2021, Tomberlin et al. 2022, Larouche et al. 2023). Our study provides a basis for designing more in-depth manipulative studies into individual-level allometry, growth, development, and reproduction of this species and complements group-level experiments that can target performance traits important to mass-rearing of this species (Muzzatti et al. 2025).

We found *G. sigillatus* instars are identifiable based on external morphology, especially late nymphal stages between when sexual characteristics first appear and the adult stage. Instars could be estimated from expert inspection and estimates validated from trait values. Head width provided the best discrimination for instar, is used to discriminate instars in other insect species (Calvo and Molina 2008) and was highly correlated with other morphological traits (**Supplementary Fig. S2**). Evaluating the utility of morphological traits depends on their intended use. For example, abdomen length is a less reliable indicator of instar or body size because the abdomen may greatly increase in length and width after eating, but may be relevant for traits associated with egg production or feeding (Fudlosid et al. 2022, Muzzatti et al. 2022). We observed that pronotum width could extend slightly after eating. We suggest using 2 or more morphometric traits in combination with wet mass to obtain the most accurate estimation of instar, especially for instars 1 to 4, as the overlap of data between close instars may lead to incorrect estimations, particularly for individuals that deviate from the mean. Knowledge of instars is useful for tracking progression of development, identifying important stages of development, and assessing inter-individual variation in a population (Bien et al. 2023).

Wet mass was highly correlated with morphological traits but was less reliable than morphology for estimating instar because there was larger variation in wet mass within an instar (**Supplementary Fig. S1**, individuals of the same instar and sex fall along the x-axis with minimal change in morphological trait values). The decoupling of wet mass and body size through instar progression has been reported across insects (Chown and Gaston 2010). One reason for this decoupling is the change in body composition from the development of sexual characteristics at later instars. For example, adult females had a lower water content than adult males that likely reflects the development of fatty tissues such as eggs and ovaries (**Table 3**). Changes in internal anatomy, such as the presence of extensive air sacs, which are necessary to support large body sizes do not increase body mass proportionally (Harrison et al. 2023). This overall phenomenon is relevant to insects farmed for food and feed because desirable nutritional qualities for a final product may not tightly correlate with body mass. That is, increases in body size may not increase the nutritional content of a cricket because its larger body size is attributed to a more extensive tracheal system required to maintain a larger body size. Thus, selection on body size and not body mass may not be beneficial for a farm aiming to increase yield or nutritional content. Crickets that are heavier may contain more tissue that contributes to the final nutritional content of the product. Our body water content estimates were higher than other Orthopterans but water content varies greatly among insect species (Hadley 1994, Woodman 2012).

Our study used instar as a proxy for developmental stage rather than time as other studies have done. Few studies have reported growth and development of *G. sigillatus* over ontogeny and these studies have used week as a proxy for age and instar (eg McFarlane 1964, Fudlosid et al. 2022, Muzzatti et al. 2022). Other studies are limited to later nymphal stages or the adult stage (eg Rapkin et al. 2018). Our data can be used to infer instar from studies reporting age in time or from wet mass or body size. A fine-scale understanding of growth allows for certain time periods that may be of interest for a farming context to be targeted (Morales-Ramos et al. 2015). For example, the fastest growth estimated by the logistic model occurred between instars 6 and 7 (approximately day 25, **Table 2**) which is when sexual morphological traits were developing (**Fig. 4A**). This transitional period may be of interest to understand patterns of maturation or responses to stressors over ontogeny that manifest at adulthood (Nowosielski and Patton 1965, Klockmann et al. 2017). We found the growth pattern of this species was well represented by a sigmoidal model, thus a sampling interval of weeks may not be able to identify or capture this critical period of growth. This further exemplifies the intensive nature of high-resolution data collection for practitioners. The timing of the inflection point in the growth curve may also change depending on the individual and the rearing conditions. Inter-individual variation in maturation in *G. sigillatus* can be high during this period of maximum growth and as sexual dimorphism emerges, even under controlled conditions. Being able to identify instar is important for ensuring sampling consistency and quantifying this inter-individual variation (much more overlap in data points in **Fig. 4A** than in earlier timepoints). For example, studies that aim to target the adult stage, such as for reproduction, will benefit from being able to identify when a cricket matures to control for the age of the cricket. Understanding the physiological basis for growth and maturation is a fundamental biological problem that encompasses allometry, drivers of optimal growth, and to what extent growth and maturation are fixed for an individual (Tennessen and Thummel 2011, Mirth et al. 2016, Meister et al. 2017, Buchanan et al. 2022). These fundamental problems can be directly translated to commercially farmed insects to optimize harvest yield as variation in maturation affects the logistics around when eggs can be collected from a breeding population, for how long, and to control for the age of the breeding females, as well as the timing of a harvest.

Our oviposition results suggest that crickets can oviposit for most of their adult life, provided females have access to oviposition substrate and mating partners. It is important to note that water alone is not a natural oviposition substrate and this could have affected female oviposition behavior. We have observed crickets reared in high density colonies laying in water and in damp paper towels even if provided peat for oviposition. However, we have no information about the oviposition preferences of this species and limited information about their mating behaviors (Bateman and MacFadyen 1999, Kindle et al. 2006). Future studies could compare oviposition with more natural substrates such as sand or soil to characterize oviposition behavior. Female crickets continued to oviposit after their partner died but it is unknown whether these eggs were fertilized although this is unlikely to be an issue for a breeding colony. Our results suggest that oviposition starts soon after mating but the delay in oviposition (median of 6 d in our study) suggests providing substrate too early would not capture oviposition from all females. As a farm may aim to provide substrate for only a few days to obtain eggs of a similar age, our results suggest delaying the provision of substrate for a week may be beneficial to capture oviposition from more females. Our results also suggest eggs can be collected repeatedly with the maximum output around 14 to 20 d.

Our data provide baseline trait values that can be used to identify problems and anticipate challenges for mass-rearing crickets. For example, optimizing rearing conditions typically involves manipulating external variables (eg temperature, density, or availability of food and water) and measuring life-history traits relating to yield (Hawkey et al. 2021). Our data can be used to evaluate the impacts of manipulating one or more variables with the goal to optimize yield and can complement further quantification of phenotypes and behaviors.

Second, it is likely there is significant variation in traits among populations or strains that have evolved through the separation of farmed populations over time. For example, our mean fecundity in a 48-h period was lower than previously reported for outbred lines of *G. sigillatus* for the same sampling period (Archer et al. 2012). We also did not find a decrease in fecundity over time (as a proxy for parental age) as previously reported for a similar time frame (30 d) (Archer et al. 2012), and longevity and resting metabolic rate differed between 2 laboratory populations of *G. sigillatus* sourced from the United States of America and Australia (Okada et al. 2011). Comparisons with more than 2 populations would facilitate our understanding of evolved variation in this species and quantify the effects of captivity. Such information would assist insect farmers in identifying whether their stock population has deviated significantly from an original source.

Third, phenotypic variation or lack thereof in farmed populations may indicate a genetic basis for patterns of variation (Nakajima and Ogura 2022). Genomics represents an untapped potential for farmed insects and our ability to link genes with fitness related phenotypes that are beneficial for farming, or to target specific genes, depends on characterizing life-history phenotypes (Nakamura et al. 2022). *Gryllodes sigillatus* has a tropical worldwide distribution including in urban areas and human habitats, which likely permits range extensions far into temperate areas through the movement of individuals for the pet food industry (Masaki 1978, Toms 1993, Bateman et al. 2005). This distribution suggests *G. sigillatus* is adaptable to a range of habitats within their environmental tolerance. Studies on inbred lines of *G. sigillatus* demonstrated that sustained inbreeding has profound negative effects on life-history traits and fitness that would affect colony maintenance, such as decreased female fecundity, decreased male calling durations, and decreased immune responses to an immunity challenge (Gershman et al. 2010, Sakaluk et al. 2019). These pedigree studies also suggest that *G. sigillatus* would be responsive to directed selection and experimental evolution. Leveraging an understanding of phenotypic and genetic diversity, there is potential to explore the development of strains optimized for mass-rearing which may also minimize variability in yields, while considering that selection on body size may not correspond with body mass or desired product qualities (Larouche et al. 2023).

Fourth, life-history data from a successful population, such as this study, can be used to compare with unsuccessful or collapsed populations to facilitate identifying the underlying causes. Disease is an important issue for mass-rearing insects from an animal welfare and food safety perspective (Fernandez-Cassi et al. 2020, Vogel et al. 2022). Viruses, parasites, and microbes that negatively affect the performance of mass-reared insects may contribute toward variability in farming yields or colony collapses (Bertola and Mutinelli 2021, Jordan and Tomberlin 2021, Herren et al. 2023). Early indicators of stress or disease that precede a potential colony collapse may be detectable by phenotypic traits. Identifying these signals is crucial for monitoring and deciding when to intervene and form the basis for training artificial intelligence models to monitor insect welfare in mass-reared contexts through changes in behavioral patterns or visual signals (Hansen et al. 2022, Wenning et al. 2022). These emerging technologies can complement genomic approaches for monitoring disease or the selection of resistant traits (Maciel-Vergara et al. 2021, Lim et al. 2024).

Life history provides a framework in which to understand variation in performance that in turn underpins yield. More generally, high resolution morphological data and validated instar estimates, such as this study, are essential for training artificial intelligence models that aim to record growth and development of individual crickets, as well as population sizes in high-density precision farming, as have been developed for other livestock (Tedeschi et al. 2021, Bao and Xie 2022). These emerging technologies facilitate researchers to scale up data processing to throughputs needed for industrial production as currently processing images to measure morphometrics, for example, is done manually (Wenning et al. 2022). Moving beyond monitoring, our understanding of insect growth and development directly informs models of animal growth that can be used to predict growth and the timing of optimal harvests (Mauritsson and Jonsson 2023). For example, life-history traits, feeding rates, and metabolic rate can feed into a Dynamic Energy Budget model to predict growth and development of an insect. These models exist for holometabolous insects but have only recently been developed for a hemimetabolous insect (Llandres et al. 2015, Klagkou et al. 2024). Overall, an integrative and collaborative approach between stakeholders will facilitate research breakthroughs in insect farming for food and feed (Robinson et al. 2024).

## Supplementary material

Supplementary material is available at *Journal of Economic Entomology* online.

toaf073_suppl_Supplementary_Figures_S1-S3_Tables_S1

## References

[CIT0001] Archer CR , Zajitschek F, Sakaluk SK, et al 2012. Sexual selection affects the evolution of lifespan and ageing in the decorated cricket *Gryllodes sigillatus*. Evolution 66:3088–3100. https://doi.org/10.1111/j.1558-5646.2012.01673.x 23025600

[CIT0002] Bao J , Xie Q. 2022. Artificial intelligence in animal farming: a systematic literature review. J. Clean Prod. 331:129956. https://doi.org/10.1016/j.jclepro.2021.129956.

[CIT0003] Bateman PW , MacFadyen DN. 1999. Mate guarding in the cricket *Gryllodes sigillatus*: influence of multiple potential partners. Ethology 105:949–957. https://doi.org/10.1046/j.1439-0310.1999.00484.x

[CIT0004] Bateman PW , Gilson LN, Ferguson JWH. 2001. Investment in mate guarding may compensate for constraints on ejaculate production in the cricket *Gryllodes sigillatus*. Ethology 107:1087–1098. https://doi.org/10.1046/j.1439-0310.2001.00756.x

[CIT0005] Bateman PW , Verburgt L, Ferguson JWH. 2005. Exposure to male song increases rate of egg development in the cricket *Gryllodes sigillatus*. Afr. Zool. 40:323–326.

[CIT0006] Bertola M , Mutinelli F. 2021. A systematic review on viruses in mass-reared edible insect species. Viruses 13:2280. https://doi.org/10.3390/v13112280 34835086 PMC8619331

[CIT0007] Biagio FP , Tamaki FK, Terra WR, et al 2009. Digestive morphophysiology of *Gryllodes sigillatus* (Orthoptera: Gryllidae). J. Insect Physiol. 55:1125–1133. https://doi.org/10.1016/j.jinsphys.2009.08.015 19715697

[CIT0008] Bien T , Alexander BH, White E, et al 2023. Sizing up spotted lanternfly nymphs for instar determination and growth allometry. PLoS One 18:e0265707. https://doi.org/10.1371/journal.pone.0265707 36730235 PMC9894384

[CIT0009] Buchanan KL , Meillère A, Jessop TS. 2022. Early life nutrition and the programming of the phenotype. In: Costantini D, Marasco V editors. Development strategies and biodiversity: Darwinian fitness and evolution in the Anthropocene. Springer International Publishing. p. 161–214.

[CIT0010] Calvo D , Molina JM. 2008. Head capsule width and instar determination for larvae of *Streblote panda* (Lepidoptera: Lasiocampidae). Ann. Entomol. Soc. Am. 101:881–886. https://doi.org/10.1093/aesa/101.5.881

[CIT0011] Chavez M. 2021. The sustainability of industrial insect mass rearing for food and feed production: zero waste goals through by-product utilization. Curr. Opin. Insect Sci. 48:44–49. https://doi.org/10.1016/j.cois.2021.09.003 34597858

[CIT0012] Chen L , Sørensen JG, Enkegaard A. 2022. Acclimation for optimisation: effects of temperature on development, reproduction and size of *Trichogramma achaeae*. Biocontrol Sci. Technol. 32:60–73. https://doi.org/10.1080/09583157.2021.1963679

[CIT0013] Chia SY , Tanga CM, Khamis FM, et al 2018. Threshold temperatures and thermal requirements of black soldier fly *Hermetia illucens*: implications for mass production. PLoS One 13:e0206097. https://doi.org/10.1371/journal.pone.0206097 30383771 PMC6211680

[CIT0014] Chown SL , Gaston KJ. 2010. Body size variation in insects: a macroecological perspective. Biol. Rev. Camb. Philos. Soc. 85:139–169. https://doi.org/10.1111/j.1469-185X.2009.00097.x. 20015316

[CIT0015] Davidowitz G. 2021. Habitat-centric versus species-centric approaches to edible insects for food and feed. Curr. Opin. Insect Sci. 48:37–43. https://doi.org/10.1016/j.cois.2021.09.006. 34601184

[CIT0016] Fernandez-Cassi X , Söderqvist K, Bakeeva A, et al 2020. Microbial communities and food safety aspects of crickets (*Acheta domesticus*) reared under controlled conditions. J. Insects Food Feed 6:429–440. https://doi.org/10.3920/jiff2019.0048

[CIT0017] Fudlosid S , Ritchie MW, Muzzatti MJ, et al 2022. Ingestion of microplastic fibres, but not microplastic beads, impacts growth rates in the tropical house cricket *Gryllodes sigillatus*. Front. Physiol. 13:871149. https://doi.org/10.3389/fphys.2022.871149 35634147 PMC9132090

[CIT0018] Gershman SN , Barnett CA, Pettinger AM, et al 2010. Inbred decorated crickets exhibit higher measures of macroparasitic immunity than outbred individuals. Heredity 105:282–289. https://doi.org/10.1038/hdy.2010.1 20125187

[CIT0019] Hadley NF. 1994. Water relations of terrestrial arthropods. Academic Press, Inc.

[CIT0020] Hansen MF , Oparaeke A, Gallagher R, et al 2022. Towards machine vision for insect welfare monitoring and behavioural insights. Front. Vet. Scie. 9:835529. https://doi.org/10.3389/fvets.2022.835529 PMC8886630 35242842

[CIT0021] Harrison JF , McKenzie EKG, Talal S, et al 2023. Air sacs are a key adaptive trait of the insect respiratory system. J. Exp. Biol. 226:jeb245712. https://doi.org/10.1242/jeb.245712 37204298

[CIT0022] Hawkey KJ , Lopez-Viso C, Brameld JM, et al 2021. Insects: a potential source of protein and other nutrients for feed and food. Annu. Rev. Anim. Biosci. 9:333–354. https://doi.org/10.1146/annurev-animal-021419-083930 33228376

[CIT0023] Herren P , Hesketh H, Meyling NV, et al 2023. Environment–host–parasite interactions in mass-reared insects. Trends Parasitol. 39:588–602. https://doi.org/10.1016/j.pt.2023.04.007. 37258342

[CIT0024] Hoc B , Noël G, Carpentier J, et al 2019. Optimization of black soldier fly (*Hermetia illucens*) artificial reproduction. PLoS One 14:e0216160. https://doi.org/10.1371/journal.pone.0216160 31039194 PMC6490921

[CIT0025] Jordan HR , Tomberlin JK. 2021. Microbial influence on reproduction, conversion, and growth of mass produced insects. Curr. Opin. Insect Sci. 48:57–63. https://doi.org/10.1016/j.cois.2021.10.001. 34655809

[CIT0026] Ketola T , Kotiaho JS. 2010. Inbreeding, energy use and sexual signaling. Evol. Ecol. 24:761–772. https://doi.org/10.1007/s10682-009-9333-1

[CIT0027] Kindle TK , Johnson KM, Ivy TM, et al 2006. Female mating frequency increases with temperature in two cricket species, *Gryllodes sigillatus* and *Acheta domesticus* (Orthoptera: Gryllidae). Can. J. Zool. 84:1345–1350. https://doi.org/10.1139/z06-127

[CIT0028] Klagkou E , Gergs A, Baden CU, et al 2024. Dynamic Energy Budget approach for modeling growth and reproduction of Neotropical stink bugs. Ecol. Modell. 493:110740. https://doi.org/10.1016/j.ecolmodel.2024.110740.

[CIT0029] Klockmann M , Kleinschmidt F, Fischer K. 2017. Carried over: heat stress in the egg stage reduces subsequent performance in a butterfly. PLoS One 12:e0180968. https://doi.org/10.1371/journal.pone.0180968 28708887 PMC5510857

[CIT0030] Larouche J , Campbell B, Hénault-Éthier L, et al 2023. The edible insect sector in Canada and the United States. Anim Front 13:16–25. https://doi.org/10.1093/af/vfad047 PMC10425141 37583805

[CIT0031] Liceaga AM , Aguilar-Toalá JE, Vallejo-Cordoba B, et al 2022. Insects as an alternative protein source. Annu. Rev. Food Sci. Technol. 13:19–34. https://doi.org/10.1146/annurev-food-052720-112443 34699254

[CIT0032] Lim FS , González-Cabrera J, Keilwagen J, et al 2024. Advancing pathogen surveillance by nanopore sequencing and genotype characterization of Acheta domesticus densovirus in mass-reared house crickets. Sci. Rep. 14:8525. https://doi.org/10.1038/s41598-024-58768-3 38609404 PMC11014933

[CIT0033] Llandres AL , Marques GM, Maino JL, et al 2015. A dynamic energy budget for the whole life-cycle of holometabolous insects. Ecol. Monogr. 85:353–371. https://doi.org/10.1890/14-0976.1

[CIT0034] Maciel-Vergara G , Jensen AB, Lecocq A, et al 2021. Diseases in edible insect rearing systems. J. Insects Food Feed 7:621–638. https://doi.org/10.3920/jiff2021.0024.

[CIT0035] Magara HJO , Niassy S, Ayieko MA, et al 2021. Edible crickets (Orthoptera) around the world: distribution, nutritional value, and other benefits—a review. Front. Nutr. 7:537915. https://doi.org/10.3389/fnut.2020.537915 33511150 PMC7835793

[CIT0036] Mamai W , Lobb LN, Bimbilé Somda NS, et al 2018. Optimization of mass-rearing methods for *Anopheles arabiensis* larval stages: effects of rearing water temperature and larval density on mosquito life-history traits. J. Econ. Entomol. 111:2383–2390. https://doi.org/10.1093/jee/toy213 30020467

[CIT0037] Masaki S. 1978. ‘Chapter 4 seasonal and latitudinal adaptations in the life cycles of crickets. In: Dingle H, editor. Evolution of insect migration and diapause. Springer US. p. 72–100.

[CIT0038] Mauritsson K , Jonsson T. 2023. A new flexible model for maintenance and feeding expenses that improves description of individual growth in insects. Sci. Rep. 13:16751. https://doi.org/10.1038/s41598-023-43743-1 37798309 PMC10556006

[CIT0039] McFarlane JE. 1964. Factors affecting growth and wing polymorphism in *Gryllodes sigillatus* (Walk.): dietary protein level and a possible effect of photoperiod. Can. J. Zool. 42:767–771. https://doi.org/10.1139/z64-074

[CIT0040] Meister H , Esperk T, Välimäki P, et al 2017. Evaluating the role and measures of juvenile growth rate: latitudinal variation in insect life histories. Oikos 126:1726–1737. https://doi.org/10.1111/oik.04233

[CIT0041] Mirth CK , Anthony Frankino W, Shingleton AW. 2016. Allometry and size control: what can studies of body size regulation teach us about the evolution of morphological scaling relationships? Curr. Opin. Insect Sci. 13:93–98. https://doi.org/10.1016/j.cois.2016.02.010. 27436558

[CIT0042] Moore MP , Martin RA. 2019. On the evolution of carry-over effects. J. Anim. Ecol. 88:1832–1844. https://doi.org/10.1111/1365-2656.13081 31402447

[CIT0045] Morales-Ramos JA , Kay S, Rojas MG, et al 2015. Morphometric analysis of instar variation in *Tenebrio molitor* (Coleoptera: Tenebrionidae). Ann. Entomol. Soc. Am. 108:146–159. https://doi.org/10.1093/aesa/sau049

[CIT0043] Morales-Ramos JA , Rojas MG, Dossey AT, et al 2020. Self-selection of food ingredients and agricultural by-products by the house cricket, *Acheta domesticus* (Orthoptera: Gryllidae): a holistic approach to develop optimized diets. PLoS One 15:e0227400. https://doi.org/10.1371/journal.pone.0227400 31978186 PMC6980616

[CIT0044] Morales-Ramos JA , Tomberlin JK, Miranda C, et al 2024. Rearing methods of four insect species intended as feed, food, and food ingredients: a review. J. Econ. Entomol. 117:1210–1224. https://doi.org/10.1093/jee/toae040 38501911

[CIT0046] Mutamiswa R , Tarusikirwa VL, Nyamukondiwa C, et al 2023. Thermal stress exposure of pupal oriental fruit fly has strong and trait-specific consequences in adult flies. Physiol. Entomol. 48:35–44. https://doi.org/10.1111/phen.12400

[CIT0047] Muzzatti MJ , McConnell E, Neave S, et al 2022. Fruitful female fecundity after feeding *Gryllodes sigillatus* (Orthoptera: Gryllidae) royal jelly. Can. Entomol. 154:e50. https://doi.org/10.4039/tce.2022.39

[CIT0048] Muzzatti MJ , Kong JD, McColville ER, et al 2025. Diet particle size influences tropical house cricket life history. J. Insects Food Feed 1–11. Online ahead of print. https://doi.org/10.1163/23524588-00001365

[CIT0049] Nakajima Y , Ogura A. 2022. Genomics and effective trait candidates of edible insects. Food Biosci. 48:101793. https://doi.org/10.1016/j.fbio.2022.101793.

[CIT0050] Nakamura T , Ylla G, Extavour CG. 2022. Genomics and genome editing techniques of crickets, an emerging model insect for biology and food science. Curr. Opin. Insect Sci. 50:100881. https://doi.org/10.1016/j.cois.2022.100881. 35123119

[CIT0051] Nowosielski JW , Patton RL. 1965. Variation in the haemolymph protein, amino acid, and lipid levels in adult house crickets, *Acheta domesticus* L., of different ages. J. Insect Physiol. 11:263–270. https://doi.org/10.1016/0022-1910(65)90074-0. 14327211

[CIT0052] Okada K , Pitchers WR, Sharma MD, et al 2011. Longevity, calling effort, and metabolic rate in two populations of cricket. Behav. Ecol. Sociobiol. 65:1773–1778. https://doi.org/10.1007/s00265-011-1185-3

[CIT0053] Pinheiro JC , Bates D. 2009. Mixed-effects models in S and S-PLUS. Springer.

[CIT0054] R Core Team. 2023. ‘Chapter R: a language and environment for statistical computing’. 4.3. R Foundation for Statistical Computing.

[CIT0055] Raheem D , Carrascosa C, Oluwole OB, et al 2019. Traditional consumption of and rearing edible insects in Africa, Asia and Europe. Crit. Rev. Food Sci. Nutr. 59:2169–2188. https://doi.org/10.1080/10408398.2018.1440191 29446643

[CIT0056] Rapkin J , Jensen K, Archer CR, et al 2018. The geometry of nutrient space–based life-history trade-offs: sex-specific effects of macronutrient intake on the trade-off between encapsulation ability and reproductive effort in decorated crickets. Am. Naturalist 191:452–474. https://doi.org/10.1086/696147 29570407

[CIT0057] Ritchie MW , Provencher JF, Allison JE, et al 2024. The digestive system of a cricket pulverizes polyethylene microplastics down to the nanoplastic scale. Environ. Pollut. 343:123168. https://doi.org/10.1016/j.envpol.2023.123168 38104765

[CIT0058] Robinson K , Duffield KR, Ramirez JL, et al 2024. MINIstock: model for INsect Inclusion in sustainable agriculture: USDA-ARS’s research approach to advancing insect meal development and inclusion in animal diets. J. Econ. Entomol. 117:1199–1209. https://doi.org/10.1093/jee/toae130 38961669

[CIT0059] Sakaluk SK , Oldzej J, Poppe CJ, et al 2019. Effects of inbreeding on life-history traits and sexual competency in decorated crickets. Anim. Behav. 155:241–248. https://doi.org/10.1016/j.anbehav.2019.05.027

[CIT0060] Schindelin J , Arganda-Carreras I, Frise E, et al 2012. Fiji: an open-source platform for biological-image analysis. Nat. Methods 9:676–682. https://doi.org/10.1038/nmeth.2019 22743772 PMC3855844

[CIT0061] Tedeschi LO , Greenwood PL, Halachmi I. 2021. Advancements in sensor technology and decision support intelligent tools to assist smart livestock farming. J. Anim. Sci. 99:skab038. https://doi.org/10.1093/jas/skab038 33550395 PMC7896629

[CIT0062] Tennessen Jason M , Thummel Carl S. 2011. Coordinating growth and maturation — insights from *Drosophila*. Curr. Biol. 21:R750–R757. https://doi.org/10.1016/j.cub.2011.06.033 21959165 PMC4353487

[CIT0063] Tomberlin JK , Picard CJ, Jordan HR, et al 2022. Government and industry investment plays crucial role in further establishment, evolution, and diversification of insect agriculture: a case example from the United States. J. Insects Food Feed 8:109–111. https://doi.org/10.3920/jiff2022.x001.

[CIT0064] Toms RB. 1993. More winged females of the cricket *Gryllodes supplicans* (Walker). S. Afr. J. Zool. 28:122–124. https://doi.org/10.1080/02541858.1993.11448304

[CIT0065] UN. 2015. Transforming our world: the 2030 agenda for sustainable development.

[CIT0066] van Huis A. 2020. Insects as food and feed, a new emerging agricultural sector: a review. J. Insects Food Feed 6:27–44. https://doi.org/10.3920/jiff2019.0017.

[CIT0072] van Huis A , Rumpold B, Maya C, et al 2021. Nutritional qualities and enhancement of edible insects. Ann. Rev. Nutr. 41:551–576. https://doi.org/10.1146/annurev-nutr-041520-010856 34186013

[CIT0068] Van Peer M , Berrens S, Coudron C, et al 2024. Towards good practices for research on *Acheta domesticus*, the house cricket. J. Insects Food Feed 10:1235–1251. https://doi.org/10.1163/23524588-00001042

[CIT0069] Vogel M , Shah PN, Voulgari-Kokota A, et al 2022. Health of the black soldier fly and house fly under mass-rearing conditions: innate immunity and the role of the microbiome. J. Insects Food Feed 8:857–878. https://doi.org/10.3920/jiff2021.0151

[CIT0070] Wenning MJ , Piotrowski T, Janzen J, et al 2022. Towards monitoring of a cricket production using instance segmentation. J. Insects Food Feed 8:763–772. https://doi.org/10.3920/jiff2021.0165.

[CIT0071] Woodman JD. 2012. Cold tolerance of the Australian spur-throated locust, *Austracris guttulosa*. J. Insect Physiol. 58:384–390. https://doi.org/10.1016/j.jinsphys.2011.12.015. 22226821

