# Supplementary material for: Growth, development, and life history of a mass-reared edible insect, Gryllodes sigillatus (Orthoptera: Gryllidae)
Source: J Econ Entomol. 2025 Apr 18;118(3):1093–103. doi: 10.1093/jee/toaf073 (PMC12167847; doi:10.1093/jee/toaf073)
Supplement: toaf073_suppl_Supplementary_Figures_S1-S3_Tables_S1 [file toaf073_suppl_supplementary_figures_s1-s3_tables_s1.docx]

**Supplementary material for Growth, development, and life history of a mass-reared edible insect, *Gryllodes sigillatus*** **(Orthoptera:** **Gryllidae)**

Jacinta D. Kong*, Marshall W. Ritchie, Émile Vadboncoeur, Heath A. MacMillan, and Susan M. Bertram

Department of Biology, Carleton University, Ottawa, Ontario, K1S 5B6, Canada

*Author for correspondence ([JacintaKong@cunet.carleton.ca](mailto:JacintaKong@cunet.carleton.ca))


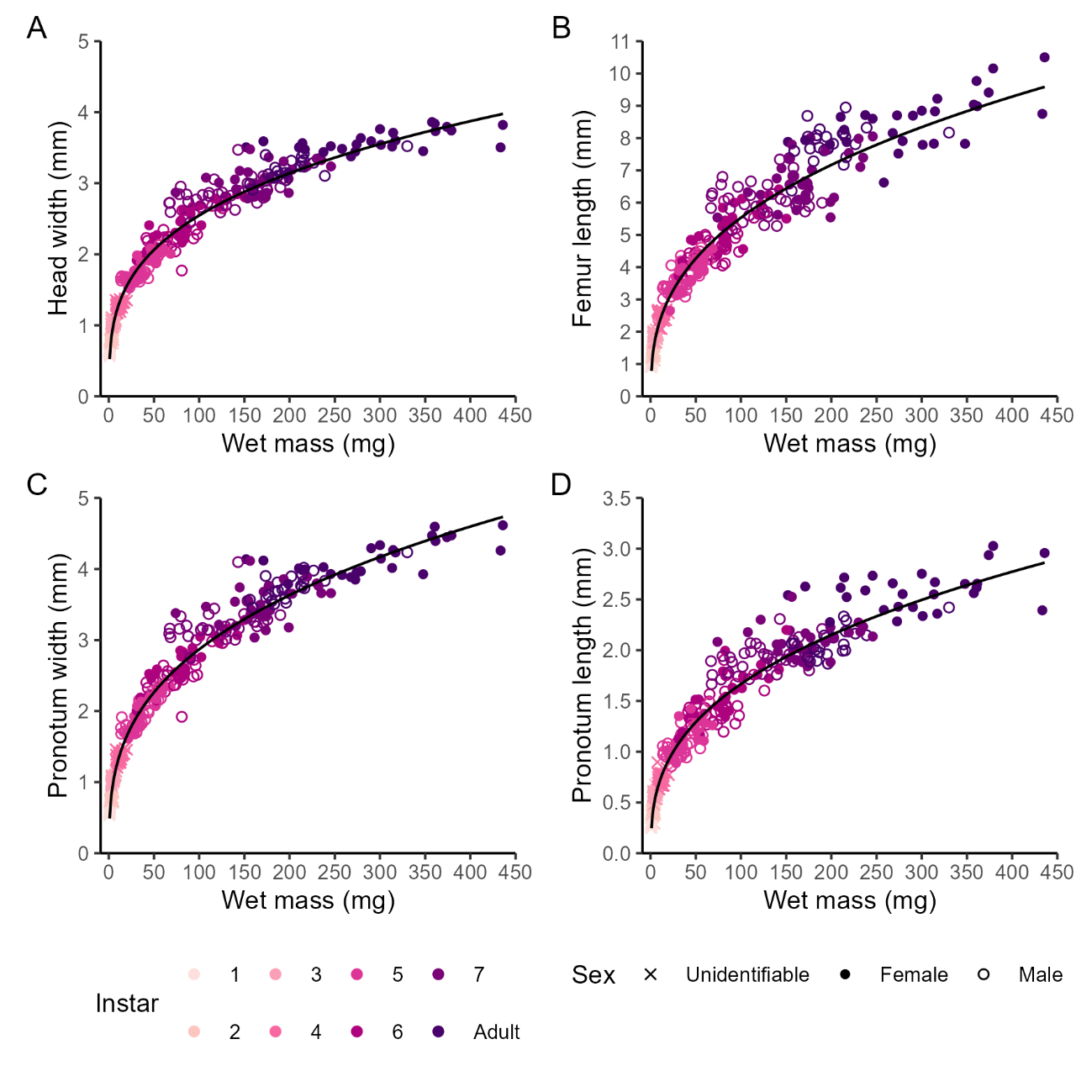


**Fig.** **S1**. A) Head width, B) Femur length, C) Pronotum width and D) Pronotum length of 355 *Gryllodes sigillatus* crickets by wet mass. Colors denote instar. Crosses denote juvenile crickets of unidentifiable sex (instars 1 – 4), closed circles denote female crickets (instars 5 – 8), and open circles denote male crickets (instars 5 – 7 & Adult). Solid black line indicates the fitted linear regression to Log_10_ transformed traits and Log_10_ transformed wet mass, pooling sex (Table S1).

**Table S1**. Regression equations and Coefficient of Determination, R^2^ (%), for the allometric relationships shown in **Fig.** S1.

| Figure panel | Trait | Equation | R^2^ (%) |
| --- | --- | --- | --- |
| Fig. S1A | Head width (mm) by wet mass (mg) | Log_10_(Head width) = 0.30 × Log_10_(Wet mass)– 0.20 | 97.26 |
| Fig. S1B | Femur length (mm) by wet mass (mg) | Log_10_(Femur length) = 0.37 × Log_10_(Wet mass)– 0.0009 | 96.67 |
| Fig. S1C | Pronotum width (mm) by wet mass (mg) | Log_10_(Pronotum width) = 0.34 × Log_10_(Wet mass)– 0.22 | 97.79 |
| Fig. S1D | Pronotum length (mm) by wet mass (mg) | Log_10_(Pronotum length) = 0.36 × Log_10_(Wet mass)– 0.51 | 96.54 |


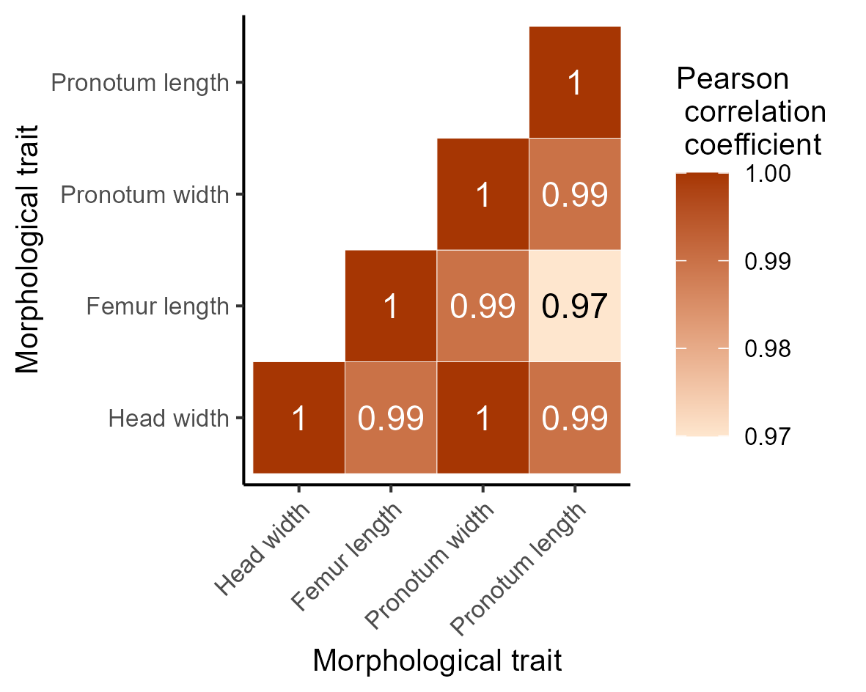


**Fig.** **S2**. Pearson correlation coefficients (colors) for pairwise correlations between morphological traits throughout ontogeny, pooling sex (n = 355 crickets).


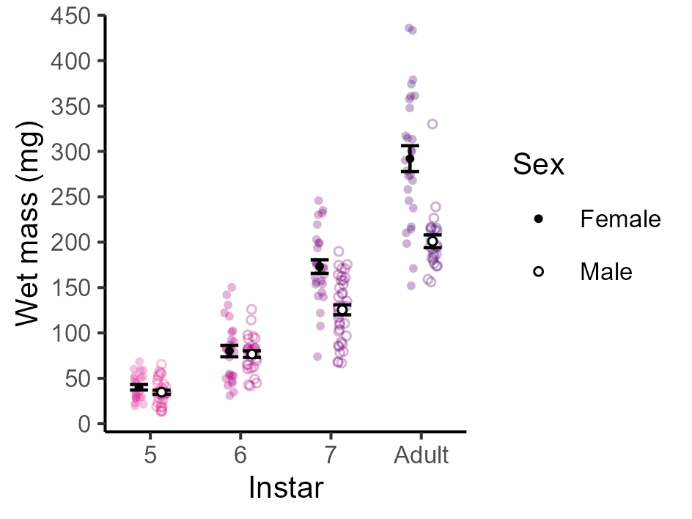


**Fig.** **S3**. Wet mass of male (open circles) and female (closed circles) *Gryllodes sigillatus* crickets (n = 231) for instars 5-7 & Adult (colors) when sex could be determined.


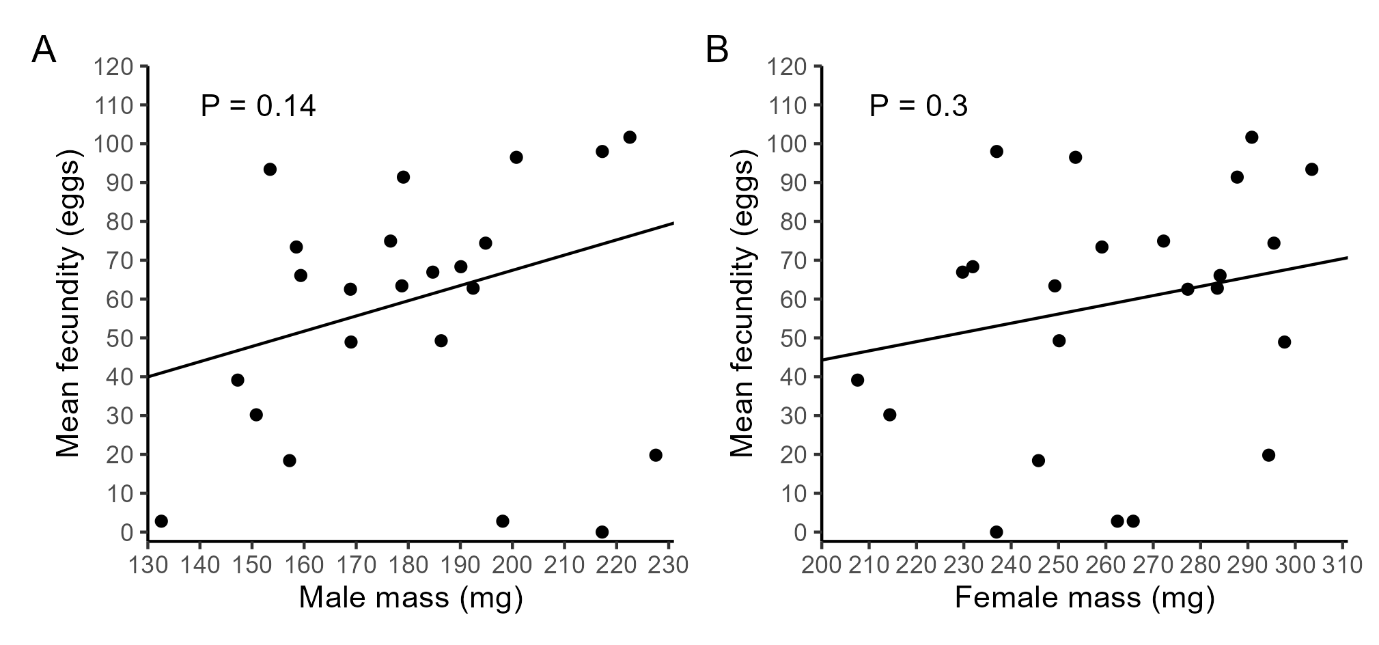


**Fig.** **S4**. Association between male adult mass (mg, **A**) and female adult mass (mg, **B**) and mean fecundity for each mating pair (points, n = 23) of *Gryllodes sigillatus* crickets. Solid line indicates the linear regression with P value shown.
